# Elective high-frequency oscillatory ventilation in preterm infants with respiratory distress syndrome: an individual patient data meta-analysis

**DOI:** 10.1186/1471-2431-9-33

**Published:** 2009-05-16

**Authors:** Filip Cools, Lisa M Askie, Martin Offringa, the Prevention of Ventilator Induced Lung Injury collaborative study Group (PreVILIG Collaboration)

**Affiliations:** 1 Department of Neonatology, Universitair Ziekenhuis Brussels, Belgium; 2 NHMRC Clinical Trials Centre, University of Sydney, Sydney, NSW, Australia; 3 Department of Pediatric Clinical Epidemiology, Academic Medical Center, Amsterdam, The Netherlands

## Abstract

**Background:**

Despite the considerable amount of evidence from randomized controlled trials and meta-analyses, uncertainty remains regarding the efficacy and safety of high-frequency oscillatory ventilation as compared to conventional ventilation in the early treatment of respiratory distress syndrome in preterm infants. This results in a wide variation in the clinical use of high-frequency oscillatory ventilation for this indication throughout the world. The reasons are an unexplained heterogeneity between trial results and a number of unanswered, clinically important questions. Do infants with different risk profiles respond differently to high-frequency oscillatory ventilation? How does the ventilation strategy affect outcomes? Does the delay – either from birth or from the moment of intubation – to the start of high-frequency oscillation modify the effect of the intervention? Instead of doing new trials, those questions can be addressed by re-analyzing the individual patient data from the existing randomized controlled trials.

**Methods/Design:**

A systematic review with meta-analysis based on individual patient data. This involves the central collection, validation and re-analysis of the original individual data from each infant included in each randomized controlled trial addressing this question.

The study objective is to estimate the effect of high-frequency oscillatory ventilation on the risk for the combined outcome of death or bronchopulmonary dysplasia or a severe adverse neurological event. In addition, it will explore whether the effect of high-frequency oscillatory ventilation differs by the infant's risk profile, defined by gestational age, intrauterine growth restriction, severity of lung disease at birth and whether or not corticosteroids were given to the mother prior to delivery. Finally, it will explore the importance of effect modifying factors such as the ventilator device, ventilation strategy and the delay to the start of high-frequency ventilation.

**Discussion:**

An international collaborative group, the PreVILIG Collaboration (Prevention of Ventilator Induced Lung Injury Group), has been formed with the investigators of the original randomized trials to conduct this systematic review. In the field of neonatology, individual patient data meta-analysis has not been used previously. Final results are expected to be available by the end of 2009.

## Background

### Clinical significance of respiratory distress syndrome in preterm infants

Prematurely born infants frequently suffer from respiratory distress syndrome (RDS). Of all infants born at a gestational age of less than 30 weeks, 90% require mechanical ventilation and almost 80% are treated with exogenous surfactant. This proportion is highest among the most immature infants [1]. Despite the advances in neonatal respiratory care, a considerable number of those infants develop chronic lung disease of prematurity, called bronchopulmonary dysplasia (BPD). Of all infants with a birth weight of less than 1500 g, 23% are oxygen dependent at the postmenstrual age of 36 weeks [2]. The risk for BPD is particularly high for the extremely preterm infant born at 26 weeks gestation or less: 44% to 74% need supplemental oxygen at 36 weeks postmenstrual age [3-6]. Bronchopulmonary dysplasia is associated with prolonged neonatal intensive care, home oxygen use, recurrent respiratory infections often requiring hospitalization, feeding difficulties with impaired growth and neurodevelopmental delay [7-9].

Although the pathogenesis of BPD is multifactorial, mechanical ventilation is one of the most important causative factors. Alveolar overexpansion secondary to high lung volume ("volutrauma") as well as alveolar injury due to repetitive alveolar recruitment-derecruitment ("atelectrauma") are pathogenetic mechanisms of "ventilation-induced lung injury" (VILI) that may lead to BPD [10]. Both phenomena occur during conventional mechanical ventilation (CV) of atelectasis-prone lungs, such as in premature infants with RDS.

In the late seventies "high-frequency oscillatory ventilation" (HFOV) was developed as a new ventilation technique, using tidal volumes smaller than anatomical dead space delivered at a very high rate of 600 to 900 per minute, thus avoiding the large volume swings seen with CV. High-frequency oscillatory ventilation can be delivered using a piston pump or an oscillating electromagnetic membrane (so called "true oscillators") that generate true negative pressure during exhalation phase or using a high-frequency flow interrupter where a Venturi-system at the exhalation valve is responsible for creating a negative pressure (high-frequency flow interruption or HFFI). HFOV proved to be an effective way to ventilate both normal and abnormal lungs [11,12] and in animal models of RDS, it caused less lung injury compared to CV [13]. Therefore, HFOV was expected to result in less mortality and less BPD, when used as the primary mode of ventilation in the treatment of RDS in preterm infants.

### Randomized trials of elective HFOV versus CV in preterm infants with RDS

The first randomized controlled trial, the multi-centre HIFI trial published in 1989, showed no benefit of HFOV for pulmonary outcomes and an increased risk of adverse neurological outcome (intracranial haemorrhage and periventricular leukomalacia) [14]. However, the trial was criticized afterwards because the HFOV strategy did not use lung volume recruitment ("optimal lung volume strategy" or OLVS) and because many centers lacked experience with this new ventilation technique. Numerous randomized trials followed over the next 15 years, most of them applying the "optimal lung volume strategy" with HFOV. However, the positive effects of HFOV found in animal experiments was not reproduced consistently in the clinical trials with preterm infants. Results differed substantially between trials, both for the benefits (reduction of BPD) as well as for the harms (increased risk of intracranial haemorrhage). Although the first Cochrane review, which was published in 1996, showed a modest benefit from HFOV over CV in terms of pulmonary outcome, HFOV did not become the standard of care in the ventilatory management of preterm infants with RDS.

### Summary of systematic reviews of aggregate data in 2008

Several meta-analyses about the effects of the early use of HFOV in preterm infants exist [15-22]. The updated Cochrane review [22] identified 15 trials including a total number of 3585 premature infants. Results of the meta-analyses showed no difference in mortality [relative risk (RR) for death at 36–37 weeks postmenstrual age or discharge: 0.98, 95% confidence interval (95% CI) 0.83–1.14] and a small but significant reduction in the risk of BPD at 36–37 weeks postmenstrual age or discharge in survivors (RR 0.89, 95% CI 0.81 – 0.99). However, there was significant heterogeneity between trials (p = 0.002, I^2 ^= 63.2%). A priori planned subgroup analyses based on the HFOV strategy – i.e. whether or not an OLVS was used – could not explain this heterogeneity, which persisted in the subgroup of trials with OLVS (p = 0.002). The review also demonstrated a non-significant trend towards an increased risk of adverse neurological events: RR for severe grade intracranial haemorrhage 1.11 (95% CI 0.95 – 1.30), and for periventricular leukomalacia 1.10 (95% CI 0.85 – 1.43). This result was moderately heterogeneous (not statistically significant, p = 0.11). In the subgroup analysis of trials not using a OLVS the risk for adverse neurological events was significantly higher [RR for severe grade intracranial haemorrhage 1.45 (95% CI 1.09–1.93), and for periventricular leukomalacia 1.64 (95% CI 1.02–2.64)], and the result was homogeneous, suggesting that a HFOV strategy aiming at low pressures and, thus, low lung volumes, may be associated with adverse neurological outcome.

Two recent meta-analyses comparing high-frequency ventilation with CV, used cumulative meta-analysis and meta-regression analysis to explore heterogeneity [19-21]. In the meta-analysis by Thome (17 trials, 3776 patients), the difference in risk of death or BPD did not reach statistical significance (OR 0.87, 95% CI 0.75 – 1.00). However, HFOV was associated with a significantly increased risk for air leak (OR 1.23, 95% CI 1.06 – 1.44). Cumulative meta-analysis suggested that the benefit from HFOV over CV decreased after the introduction in clinical trials of surfactant replacement therapy and of a more "lung protective" conventional ventilation strategy [19,20].

Despite all these additional subgroup analyses, cumulative meta-analyses and meta-regression analyses, heterogeneity between trial results remains largely unexplained. Because several important questions remain unanswered, the available evidence is unable to guide clinicians in making decisions about patient care: Is HFOV equally effective in all preterm infants with RDS or is it possible to identify certain subgroups of infants with specific risk profiles which benefit more or less from HFOV than others? To what extent does the ventilation strategy, both for HFOV and for CV, affect the difference in outcome between both ventilation modes? What is the influence of the delay between birth and the start of HFOV on the lung protective effects of HFOV in preterm infants? Hence, the controversy whether or not we should use HFOV as the primary mode of ventilation in preterm infants with RDS remains [23].

The aim of this project is to extend the reviews based on aggregate data by collecting and reanalyzing the original data on each individual infant in each trial ("individual patient data" or IPD meta-analysis) to help answer these remaining questions. The use of IPD will allow for more appropriate and flexible analyses of both subgroups and outcomes. This unique reanalysis will provide more detailed information, thereby improving the applicability of the evidence in clinical practice.

### Limitations of the reviews using published, aggregate data

• Published aggregate data is variable in outcome definition, e.g. for important outcomes such as "bronchopulmonary dysplasia", or in outcome reporting, thereby making pooling of trial results difficult or sometimes impossible.

• The information from the published aggregate data required for the subgroup analyses based on ventilator device or ventilation strategy is often imprecise (e.g. using wide ranges of settings) or absent. In addition, some trials cannot be included in those subgroup analyses, because they used more than one type of high-frequency ventilator (e.g. high-frequency oscillator and high-frequency flow interrupter) [24,25], but only report on outcomes for the whole study population.

• It is unclear whether some preterm infants benefit more or less from HFOV than others, based on their clinical characteristics such as the gestational age at birth, the severity of lung disease, or the intra-uterine growth. Only a few trials report outcomes separately for different groups of preterm infants, thereby defining the groups either by gestational age, but using different cut-off points [24,26], or by birth weight [27].

• It remains uncertain whether the delay between birth and the start of HFOV, or the time a premature infant was exposed to conventional ventilation prior to HFOV (i.e. the delay between the start of mechanical ventilation and the start of HFOV) are important factors in determining outcome with HFOV. For several trials, this information is not provided in the published aggregate data. Other trials have a very wide range of timing within their study population, making the information less useful for subgroup analyses. So, it is difficult to make recommendations with regard to the optimal timing of HFOV.

### Ways of overcoming these limitations by using individual patient data

• Obtaining individual patient data from each trial will improve the quality of the data. It will allow direct assessment of patient eligibility for inclusion in the trial and analysis of all randomized infants on an intention-to-treat basis. Importantly, it will allow redefining patient characteristics and outcomes uniformly across trials, resulting in more accurate pooled estimates of effects. For example, bronchopulmonary dysplasia can be defined at the same time point (e.g. 36 weeks' postmenstrual age) using the same criteria (e.g. oxygen dependency) for all trials.

• For certain trials, it might be possible to obtain information on measured, but previously unreported outcomes. For example, older trials report only on the "original" definition of BPD, i.e. oxygen dependency at 28 days postnatal age. However, if in those trials the last day of oxygen requirement was recorded for each infant, BPD at 36 weeks postmenstrual age can be calculated. Furthermore, trialists may be able to provide missing outcome data.

• Through more detailed information from the trial protocol on the one hand and the use of information at the individual patient level on the other hand, the ventilation strategy which was used in a trial, both for HFOV and for CV, can be assessed more accurately. Furthermore, more detailed information can be obtained regarding the timing of HFOV (i.e. delay between birth and randomization, delay between intubation and randomization). This will improve the quality of the subgroup analyses based on ventilation strategy and timing, and it will allow using those variable as co-variates in multi-variate regression analyses.

• Importantly, subgroup analyses can be done for a number of patient risk factors using the individual study infant's risk data rather than the average risk of the whole study population. This will allow investigating the effect of HFOV in certain subgroups of preterm infants (e.g. extremely preterm infants versus less preterm infants, small-for-gestational-age infants versus appropriate-for-gestational-age infants).

• Individual patient data will allow doing time-to-event pooled analyses for outcomes such as death, duration of mechanical ventilation and oxygen dependency.

## Methods and design

### Objectives

The main questions to be addressed by this study are:

• Does high-frequency ventilation, compared to conventional ventilation, offer a clinically important benefit in terms of more survival without BPD, without increasing the risk for adverse neurological events such as severe intracranial haemorrhage or periventricular leukomalacia, when used as the primary mode of ventilation in preterm infants with respiratory distress syndrome?

• Do the effects of HFOV differ according to the risk profile of the patient in terms of lung immaturity (gestational age at birth, antenatal corticosteroids or not), fetal growth (birth weight for gestational age), severity of lung disease (index of respiratory failure, chorioamnionitis), the timing of HFOV (delay between birth/intubation and randomization).

• How do the following intervention-related factors and co-interventions modify the treatment effect: high-frequency ventilator device and/or settings, ventilation strategy ("optimal lung volume recruitment" or not for HFOV, "lung protective strategy" or not for CV), exogenous surfactant therapy (timing of first dose, number of doses), postnatal treatment with corticosteroids.

### Methods

The method will be a systematic review of randomized controlled trials based on **individual patient data**. This means that for each infant randomized in each of the available trials, a predefined number of data items regarding baseline characteristics, experimental and control intervention, co-interventions and neonatal outcomes will be collected, stored in a central database, re-analyzed and pooled in a meta-analysis.

### Identifying studies

The trials included in the updated Cochrane review of aggregate data about HFOV [22] will be included in the IPD analysis. The search will be repeated using the same search strategy and databases (Medline, Embase, Cochrane Controlled Trial Register) to find more recent randomized controlled trials. Furthermore, experts in the field and trialists will be asked to report on other unfound or unpublished trials.

### Inclusion and exclusion criteria for studies

The inclusion and exclusion criteria for the types of study design, participants and interventions are listed below. Eligibility of trials will be assessed independently and unblinded for author and journal by two members of the Secretariat. Any differences in opinion regarding eligibility will be resolved by discussion. If individual patient data are unavailable from an available trial, the trial will still be included in the review and aggregate data will be used for sensitivity analyses.

#### a. Study design

Studies will be included if they were randomized controlled trials. Studies using quasi-randomization will be excluded.

#### b. Participants

Studies will be included if participants were preterm (< 35 weeks gestational age) or low birth weight (< 2000 g birth weight) infants with pulmonary dysfunction due to respiratory distress syndrome who were considered to require assisted ventilation.

#### c. Intervention

Studies will be included if the intervention was elective high-frequency oscillatory ventilation compared with conventional ventilation. HFOV could be delivered either with a true oscillator using a piston pump or an oscillating electromagnetic membrane, or with a high-frequency flow interrupter using a Venturi-system. The intervention is classified as "elective" if infants were randomized early in the course of their disease soon after mechanical ventilation was commenced, meaning that HFOV was used as the primary mode of ventilation. In contrast, trials are classified as "rescue" when patients were randomized only after meeting certain failure criteria on conventional ventilation. Those "rescue" trials will be excluded from the review. The control intervention may be any type of conventional, i.e. "tidal", time-cycled ventilation: pressure- or volume controlled, synchronized or not, with or without pressure-support, with or without volume-guarantee.

### Data management

A new set of pre-specified and clearly defined variables (both for patient-level and trial-level factors as well as for outcomes) and a newly developed coding system will be used. An Excell spreadsheet is designed for the data collection. Trialists are allowed to provide the individual patient data in any format. Data transformation to the new format and coding system will be done by the trialist himself or by the PreVILIG investigators' team. The individual patient data will be de-identified by the original investigators before they are sent to the PreVILIG Data Management Team. It will not include any patient identifying information such as names or addresses. Only authorized personnel (members of the PreVILIG Secretariat or Data Management Team) will have access to the data. However, Collaborators will continue to have control over how data from their trial is used. The individual patient data will be recoded as required and stored in a custom designed database. The data will not be used for any other purpose without permission of the collaborators.

The data will be checked with respect to range, internal consistency, consistency with published reports and missing items. Trial details such as randomization methods and intervention details will be crosschecked against published reports, trial protocols and data collection sheets. Inconsistencies or missing data will be discussed with the individual trialists and attempts will be made to resolve any problems by consensus. Each trial will be analyzed individually, and the resulting analyses and trial data will be sent to the trialists for verification.

### Data items requested from trialists

During a Collaborative Group Meeting, a list of data items to collect for each infant was proposed by the Secretariat to the PreVILIG collaborators. After discussion, a final list of data items and a recoding system for each of the variables was compiled. More detailed definitions of data items listed below can be found in Table 1. Details of the suggested coding system can be found in the Additional File *PreVILIG Coding System*.

**Table 1:** Definitions for patient characteristics at enrolment, data on trial interventions and co-interventions, and neonatal outcomes.

**Variable**	**Definition**
**Patient-level information on characteristics at enrolment**	

Complete course of antenatal corticosteroids	A course of at least 2 doses started > 48 hours before delivery or completed > 24 hours before delivery
Antenatal corticosteroids, any treatment	Any antenatal treatment with corticosteroids, regardless of the interval with delivery
Chorioamnionitis	As defined in the trial
Oxygenation index (OI)	OI = MAP × F_i_O_2 _× 100/*P*_a_O_2 _With
	MAP = mean airway pressure
	F_i_O_2 _= fractional inspired oxygen
	*P*_a_O_2 _= partial arterial oxygen tension

**Patient-level information on trial intervention and co-interventions**	

MAP at stabilization	Mean airway pressure in the first 24 hours after randomization.
F_i_O_2 _at stabilization	Fractional inspired oxygen in the first 24 hours after randomization.
Postnatal corticosteroids	Postnatal treatment with corticosteroids for the prevention or treatment of bronchopulmonary dysplasia
Early administration of cyclo-oxygenase inhibitor	Administration of indomethacin or ibuprofen in the first 24 hours of life in order to prevent patent ductus arteriosus or intracranial haemorrhage

**Neonatal outcomes**	

Death	Death before discharge from neonatal intensive care unit (NICU)
BPD at 36 weeks PMA	Oxygen therapy at the postmenstrual age of 36 weeks
Severe adverse neurological event	Intracranial haemorrhage grade 3 or 4, and/or cystic periventricular leukomalacia
Gross pulmonary air leak	Pneumothorax and/or pneumomediastinum and/or pneumopericardium
PDA requiring treatment	Patent ductus arteriosus requiring treatment with non-steroidal anti-inflammatory drugs or surgery
Any pulmonary air leak	Gross pulmonary air leak and/or pulmonary interstitial emphysema (PIE)
Neurological disability	Motor and/or cognitive and/or auditory and/or visual impairment

#### a. Trial-level information

• Informed consent

• Dates trial opened and closed to accrual

• Total number of patients randomized

• Method of random allocation

• Stratification factors used

• Methods of allocation concealment

• Blinding of outcome assessment

• Ventilator management in experimental arm (HFOV): ventilator device, frequency and inspiratory-to-expiratory-time ratio (*t*_I_:*t*_E_), initial settings of mean airway pressure (MAP), strategy of lung volume recruitment and stabilization on HFOV, target value for fractional inspired oxygen concentration (F_i_O_2_) at stabilization

• Ventilator management in control arm (CV): ventilator device, synchronization, initial settings of peak inspiratory pressure (PIP), end expiratory pressure (PEEP), rate and inspiratory time, target tidal volume

• Target values for arterial carbon dioxide tension (*P*_a_CO_2_) during mechanical ventilation

• Cross over to alternative ventilation mode after meeting predefined failure criteria, definition of failure criteria

• Weaning protocol for HFOV: HFOV until extubation/switch to CV when meeting certain criteria/switch to CV after a preset number of days on HFOV

• Protocol for surfactant replacement therapy: criteria for first dose, timing of first dose, type of surfactant

• Criteria for postnatal treatment with corticosteroids for the prevention or treatment of BPD

#### b. Patient-level information: characteristics at study entry

• Unique identifier coded for anonymity

• Gestational age at birth

• Birth weight

• Sex

• Apgar score after 5 minutes

• Postnatal age at intubation

• Postnatal age at randomization

• Prenatal corticosteroids therapy (complete course or any steroids administered)

• Presence of chorioamnionitis (as defined in the trial)

• Severity of lung disease at study entry: partial arterial oxygen tension (*P*_a_O_2_), F_i_O_2_, MAP before/at randomization

• Multiple birth and rank order of birth

#### c. Patient-level information: data on experimental and control intervention and co-interventions

For all infants:

• F_i_O_2 _and MAP at stabilization on mechanical ventilation^¶^

• Partial arterial carbon dioxide tension (*P*_a_CO_2_) during the first 72 hours^#^

• Surfactant replacement therapy

• Type of surfactant

• Postnatal age at first dose of surfactant

• Number of surfactant doses

• Early administration of cyclo-oxygenase inhibitor (indomethacin or ibuprofen)

• Postnatal treatment with corticosteroids for the prevention or treatment of BPD

• Postnatal age at start of postnatal corticosteroid treatment

##### For infants in the experimental arm (HFOV)

• Specific make of HFOV ventilator

• Setting of high-frequency rate in the first 24 hours^¶^

##### For infants in the control arm (CV)

• Specific make of ventilator

• Synchronization of ventilation to spontaneous respiration or not

• Ventilator rate during the first 72 hours^#^

• PEEP during the first 72 hours^#^

• PIP during the first 72 hours^#^

#### d. Patient-level information: data on neonatal outcome

• Death before discharge and age at death

• Postnatal age at final extubation

• Total number of days on mechanical ventilation

• Postnatal age at last day of nasal continuous positive airway pressure (CPAP)

• Postnatal age at last day of oxygen therapy

• Gross pulmonary air leak (pneumothorax, pneumomediastinum, pneumpericardium)

• Pulmonary interstitial emphysema

• Worst grade of intracranial haemorrhage^¶¶^

• Cystic periventricular leukomalacia

• Worst stage of retinopathy of prematurity (ROP)^##^

• Patent ductus arteriosus requiring treatment (non-steroidal anti-inflammatory drugs or surgical ligation)

• Patent ductus arteriosus requiring surgical ligation

• Postnatal age at discharge from neonatal intensive care unit

• Cerebral palsy at 1–2 years corrected age + corrected age at assessment

• Neurological disability at 1–2 years corrected age + corrected age at assessment

• Cerebral palsy at 5–8 years corrected age + corrected age at assessment

• Neurological disability at 5–8 years corrected age + corrected age at assessment

^¶ ^All recorded values in the first 24 hours will be collected, with a maximum of 4 values.

^# ^All recorded values in the first 72 hours will be collected, with a maximum of 7 values.

^¶¶ ^Grading of intracranial haemorrhage (grade 1 – 4) according to Papile [28]

^## ^Staging of ROP according to International Classification Committee [29]

### Planned analyses

The section below contains a summary of the planned analyses. A detailed analysis plan will be discussed and agreed upon by all the PreVILIG Collaborators prior to any data analysis.

Analysis will aim to be of all infants ever randomized and will be based on intention to treat. In the main analysis a two-stage approach will be taken: outcomes will be analyzed in their original trial and then these separate results will be combined in a meta-analysis to give an overall measure of effect. Both a fixed effect and a random effects model will be used. The assumption of homogeneity will be tested using the chi squared test. The I^2 ^statistic will also be used to assess consistency of results.

If, for a certain outcome, a trial has less than 80% of the complete trial data presented in individual patient data, the trial will not be included in the meta-analysis for that specific outcome.

#### 1. Outcomes to be analyzed

The main analyses comparing the effect of high-frequency ventilation with conventional ventilation will be undertaken for all outcomes listed below. The planned subgroup and sensitivity analyses will be restricted to the main outcomes only, or to the main outcomes plus a few key additional outcomes. Definitions of outcomes are listed in Table 1.

##### a. Main outcomes

• Death or BPD at 36 weeks' postmenstrual age

• Death or severe adverse neurological event

• Death or BPD at 36 weeks' postmenstrual age or severe adverse neurological event

##### b. Secondary outcomes

• death before discharge

• postnatal age at final extubation

• total number of days of mechanical ventilation

• postnatal age at last day of nasal CPAP

• postnatal age at last day of oxygen therapy

• Gross pulmonary air leak

• Any pulmonary air leak

• Severe intracranial haemorrhage (grade 3 or 4)

• Cystic periventricular leukomalacia

• Retinopathy of prematurity (stage 2 or more)

• Cross-over from assigned mode to alternative mode of ventilation after meeting trial-specific failure criteria

• Patent ductus arteriosus requiring treatment

• Patent ductus arteriosus requiring surgical ligation

• Postnatal age at discharge from NICU

• cerebral palsy at 1–2 years corrected age

• Neurological disability at 1–2 years corrected age

• Cerebral palsy at 5–8 years corrected age

• Neurological disability at 5–8 years corrected age

#### 2. Planned subgroup analyses

##### a. Trial-level characteristics

The effect of HFOV compared with CV may vary across trials because different types of ventilators or different ventilation strategies were used. To explore this further, analyses are planned whereby trials are grouped according to the characteristics of the ventilator or according to the ventilation strategy which was used.

###### (i) type of HFOV device

1. Trials will be grouped according to the characteristics of the exhalation phase into "true oscillators" using either a piston pump or an electromagnetic membrane (Hummingbird, SM 3100(A), Stephan, Dufour, SLE) and "flow interrupters" using a Venturi system (Dräger Babylog, Infant Star).

2. Trials will be grouped according to the specific make of HFOV ventilator.

###### (ii) HFOV strategy

Trials will be grouped into those that used an *"optimal lung volume strategy" (OLVS) *with HFOV and those that did not. An "optimal lung volume strategy" means that during the initial phase of stabilization on HFOV, a higher mean distending pressure than used during CV is used in order to recruit collapsed lung alveoli. Effective alveolar recruitment is reflected by a decrease in oxygen requirement because of a decrease in intrapulmonary right-to-left shunting. Ideally, oxygen requirement is expected to become 21% (room air) once all alveoli are optimally recruited and ventilated.

A trial will be considered as being an OLVS-trial if both of the following elements are present in the trial protocol:

1. an initial MAP with HFOV which is 1 to 2 cmH_2_O higher than with CV,

2. the preferential weaning of F_i_O_2 _before weaning of MAP,

###### (iii) CV strategy

Trials will be grouped into those that used a *"lung protective ventilatory strategy" (LPVS) *and those that did not. Conventional ventilation strategy will be considered as being "lung protective" if 1) atelectrauma was avoided by using adequate PEEP (4 cmH_2_O or higher), 2) volutrauma was avoided by targeting high-normal *P*_a_CO_2_-values (> 40 mmHg), limiting tidal volume (7 mL/kg or less) or, if tidal volume was not measured, using high ventilator rates (60/min or higher), and 3) spontaneous expiration by the infant during the inspiratory phase of the ventilator has been avoided by using a short inspiratory time (< 0.50 sec).

Trials will be considered as "LPVS"-trials if 3 out of the 5 following criteria are mentioned in the trial protocol:

1. PEEP of 4 cmH_2_O or higher

2. inspiratory time of less than 0.5 sec

3. initial ventilator rate of 60/minute or higher

4. P_a_CO_2 _target range with lower limit of 40 mmHg or higher

5. tidal volume target range with upper limit of 7 ml/kg or less

##### b. Patient-level characteristics

The effect of HFOV compared with CV may be different depending on the risk profile of the infant. The subgroup analyses described below will address the question whether there any particular types of preterm infants who benefit more or less from HFOV. The analyses will take into account each individual infant's own characteristics, rather than relying on summary measures of the "average" risk profile of the whole study population for a specific trial. The subgroup analyses will be restricted to the main outcomes plus 3 additional outcomes ("gross pulmonary airleak", "severe intracranial haemorrhage", and "postnatal age of last day of oxygen therapy").

The following criteria will be used to define the risk profile of the preterm infant:

###### (i) Gestational age at birth

Extremely preterm infants are at high risk of developing BPD and could therefore theoretically benefit more from HFOV on pulmonary outcomes than infants at lower risk. On the other hand, they are also at higher risk of having an intracranial haemorrhage or cerebral white matter damage. So, the final outcome could well be worse if HFOV is associated with an increased risk of adverse neurological outcome.

To determine whether HFOV offers a clinically relevant benefit in certain gestational age groups, subgroup analyses will be performed according to gestational age at birth as follows:

▪ < 26 weeks (up to 25 weeks and 6 days)

▪ 26 – 28 weeks (from 26 weeks up to 28 weeks and 6 days)

▪ 29 – 31 weeks (from 29 weeks up to 31 weeks and 6 days)

▪ 32 weeks and more

###### (ii) Severity of lung disease at onset

The severity of respiratory failure not only depends on the degree of immaturity of the infant at birth, but is also affected by factors such as the antenatal administration of corticosteroids, or the presence of chorioamnionitis and/or perinatal asphyxia.

To determine whether HFOV has more effect in infants with more severe lung disease at birth, infants will be grouped according to their oxygenation index at trial entry. The oxygenation index will be calculated for each individual infant as follows: OI = F_i_O_2 _× MAP × 100/P_a_O_2_. Three subgroups of severity of lung disease will be defined [30].

• Mild respiratory disease: OI < 4

• Moderate respiratory disease: OI 4 – 9

• Severe respiratory disease: OI > 9

###### (iii) Birth weight for gestational age

Intrauterine growth restriction is an important prenatal risk factor for BPD in very preterm infants [31]. Being at higher risk, growth restricted infants may benefit more from HFOV than other infants. Therefore, a subgroup analysis will be done for infants who are small-for-gestational age (SGA) and infants who are not SGA. Infants will be categorized within each trial, first using the trial's own definition, and secondly, using a new PreVILIG definition (i.e.: a birth weight below the 10^th ^percentile for gestational age according to their sex, using recent reference data for birth weight for premature infants)[32].

###### (iv) Antenatal administration of corticosteroids

An intervention that has changed the outcome of preterm infants dramatically is the antenatal administration of corticosteroids. It has proven to decrease the risk of death, severe IRDS and intracranial haemorrhage [33]. Therefore infants will be grouped according to whether or not they received corticosteroids antenatally. Depending on the availability of information from the trials, subgroup analyses will be done for:

1) infants who received any corticosteroids antenatally,

2) infants who received a complete course of antenatal corticosteroids

3) infants who received a complete course of antenatal corticosteroids with the last dose within 7 days before birth.

###### (iv) Timing of commencement of HFOV

Animal experiments suggest that HFOV is most successful in protecting the lungs if applied very early in the course of the disease and with minimal prior conventional ventilation. In order to differentiate between the importance of the postnatal age per se and the importance of the period of CV prior to the start of HFOV, two subgroup analyses are planned: a first subgroup analysis based on the delay between birth and randomization ("postnatal age" as effect modifying factor) and a second one based on the delay between intubation and randomization ("period of CV prior to HFOV" as effect modifying factor). Subgroups will be defined as follows:

• Delay < 1 hour

• Delay 1 – 4 hours

• Delay > 4 hours

All subgroup analyses based on patient characteristics will be performed in 2 stages. First, subgroups are analyzed within their own trial. Then, results of those analyses are pooled across trials to calculate a summary effect for each risk stratum. The subgroup analyses will be done for the main outcomes only.

#### 3) Planned sensitivity analyses

*a) *To assess whether the results are robust to trial design and quality, the following sensitivity analyses will be performed:

• Exclusion of trials with small sample size (< 100 study patients)

• Exclusion of trials with a high cross-over rate (20% or more of randomized patients in at least one treatment arm)

• Exclusion of trials without blinding of outcome assessment for outcomes such as severe intracranial haemorrhage, cystic periventricular leukomalacia and cerebral palsy

*b) *To assess whether the results are robust to different methods of analysis, the following sensitivity analyses will be performed:

• Comparison of analyses using fixed effect model and random effects model

• Comparison of analyses using IPD only and analyses using IPD and aggregate data if IPD are unavailable

*c) *To assess whether the results are robust to the inclusion or exclusion of trials with missing IPD, a sensitivity analysis will be done where IPD will be combined with aggregate data from trials with missing IPD.

#### 4) Additional analyses

Secondary, one-stage analyses will be conducted which will explore the relation between a number of trial-level and patient-level factors as co-variates and the effect of HFOV as the outcome. The influence of ventilation strategy, both for HFOV and for CV, will be further explored using this one-stage approach. A multi-variate modelling approach will be used including "trial" as a co-variate and taking into account possible interactions between different variables. Hence, predictive models for successful outcome can be developed and tested in a large study population.

### Ethical considerations

Informed consent has been given by the participants previously, at enrolment in the original trial. In this project, the patient data will not be used for any other purpose than the one in the original trial. This study is retrospective, meaning that no new patient data will be generated. Therefore, informed consent specifically for this project was not considered necessary. The individual patient data are made available through an agreement between all trialists of the Collaborative Group and the members of the Secretariat. The trialists remain the custodian of their original individual trial data at all times.

### Project management

For the purpose of this project, an international Collaborative Group, the PreVILIG Collaboration, is being formed. The Collaboration consists of 3 groups with specific responsibilities and tasks:

#### a. The Secretariat

The Secretariat is the steering group which is responsible for the project's management decisions and the daily management of the Collaboration. Its tasks are to design the project's protocol and analysis plan, organize the Collaborative Group Meetings and act as a liaison between all the members of the Collaborative Group. Membership: F Cools^1 ^(project coordinator), M Offringa^2 ^(chair), L Askie^3 ^(responsible for Data Management Team), D Henderson-Smart^4^. The Secretariat will meet once every 2 to 4 months, usually by teleconference.

^1 ^Department of Neonatology, Universitair Ziekenhuis Brussel, Brussels, Belgium;

^2 ^Department of Pediatric Clinical Epidemiology, Emma Children's University Hospital, Amsterdam, The Netherlands

^3 ^NHMRC Clinical Trials Centre, University of Sydney, Sydney, Australia

^4 ^Centre for Perinatal Health Services Research, University of Sydney, Sydney, Australia;

#### b. Trialist Group

Members of the Trialist Group will be representatives of the eligible trials. For each trial, the first author will be invited to become a member of the Collaboration. If considered necessary or if there was no response from the first author, other investigators from the trials may be contacted (data manager, statistician). In order to keep the Trialists Group updated, authors of new trials or previously unknown trials may be contacted and invited to join the Collaboration in the course of the project.

#### c. Advisory Group

Members of the Advisory Group are experts in various fields of the project, such as neonatal pulmonary mechanics and neonatal high-frequency ventilator performance (J Pillow^1^), IPD meta-analysis methodology and statistics (L Stewart^2^, C Bollen^3^), neonatal research and clinical epidemiology (R Soll^4^). In addition, the project is closely linked to the Cochrane Collaboration (R Soll, Coordinating Editor of the Neonatal Collaborative Review Group). Members of the Advisory Group will be consulted from time to time on a "on demand" basis and usually by e-mail or teleconference.

^1 ^School of Women's and Infants' Health, University of Western Australia, Perth, Australia.

^2 ^Centre for Reviews and Dissemination, University of York, UK

^3 ^Pediatric Intensive Care Unit, UMC Utrecht, The Netherlands

^4 ^Department of Pediatrics, University of Vermont College of Medicine, USA

#### d. Data Management Centre

The data management centre will be located at the NHMRC Clinical Trials Centre in Sydney, Australia, and will be coordinated by L Askie, member of the Secretariat. It will be responsible for the storage and analyses of the project data.

#### d. Collaborative Group Meetings

Collaborative group meetings will be organized on a regular basis. The members of all 3 groups will be invited to attend those meetings. The meetings will be scheduled, if possible, during international neonatal or paediatric conferences, such as the Annual Meeting of the Pediatric Academic Societies in the USA. During those meetings, various aspects of the project will be discussed with all the collaborators, such the project's design and conduct, the analysis plan, and the interpretation and reporting of the results. The final Collaborators' meeting is scheduled for May 2009 in Baltimore.

### Funding

Unrestricted research grants have been kindly provided by the companies Dräger and Nestlé to support the collection of the individual patient data by the original investigators and to organize the Collaborators' meetings. The companies are not involved in any other aspect of the project, such as the design of the project's protocol and analysis plan, the collection and the analyses of the project's data, or the interpretation and the publication of the study results.

### Publication policy

The study results will be presented in a Collaborators' meeting for discussion with all the collaborators. The main manuscript will be prepared by the project coordinator and circulated to the other members of the Secretariat for revision. The revised manuscript will be circulated to the members of the Trialist Group and Advisory Group for comment before publication. Publications will be authored in the name of the PreVILIG Collaboration as follows: F Cools, L Askie, and M Offringa for the Prevention of Ventilator Induced Lung Injury Collaborative Group (PreVILIG).

## Discussion

Despite the considerable amount of evidence about the efficacy and safety of HFOV as compared with CV as the primary mode of mechanical ventilation in preterm infants with respiratory distress syndrome, there is a wide variation in the use of HFOV in clinical practice because important questions have remained unanswered. Should we start HFOV in every preterm infant with RDS, or are there certain types of preterm infants who benefit more or less from HFOV than others? How do ventilation strategies, both of HFOV and CV, affect outcome? Is it important to start HFOV as soon as possible after birth or to limit as much as possible the time the infant is exposed to CV?

The currently available meta-analyses of randomized controlled trials are unable to answer those questions because their methodology is based on aggregate data, which has certain limitations. To overcome those limitations, a meta-analysis based on individual patient data instead of aggregate data will be conducted. This approach will improve substantially the quality of the data and will allow for more flexible analysis of both subgroups and outcomes.

In 2006, an international Collaboration (PreVILIG) was started to undertake this systematic review. Submission of the data by the participating Collaborators has been completed in November 2008 and results will be ready by August 2009. The main publication is expected in late 2009.

## Competing interests

The PreVILIG project received an unrestricted research grant from the company Dräger Int., manufacturer of a mechanical ventilator delivering both high-frequency ventilation as well as conventional ventilation (Babylog 8000 (plus)). The company Dräger was not involved in the development of the project protocol or data analysis plan, or in the collection of the trial data. Furthermore, Dräger will not have any input in the data analysis and interpretation, the preparation of the manuscripts, or the decision to publish.

## Authors' contributions

FC conceived the PreVILIG Collaboration, participated in the design, coordinated the management of the study, and drafted the manuscript. LA and MO conceived the PreVILIG study, supervised the design of the study and the statistical analysis, and edited the manuscript. All members of the PreVILIG Collaboration contributed to the design of the study, read and approved the final manuscript. The Trialist Group provided the original trial data.

## Pre-publication history

The pre-publication history for this paper can be accessed here:



## References

[B1] Horbar JD, Carpenter JH, Buzas J, Soll RF, Suresh G, Bracken MB, Leviton LC, Plsek PE, Sinclair JC, for the members of the Vermont Oxford Network (2004). Timing of initial surfactant treatment for infants 23 to 29 weeks' gestation: is routine practice evidence based?. Pediatrics.

[B2] Lemons JA, Bauer CR, Oh W, Korones SB, Papile LA, Stoll BJ, Verter J, Temprosa M, Wright LL, Ehrenkranz RA, Fanaroff AA, Stark A, Carlo W, Tyson JE, Donovan EF, Shankaran S, Stevenson DK, for the NICHD Neonatal Research Network (2001). Very low birth weight outcomes of the National Institute of Child Health and Human Development Neonatal Research Network, January 1995 through December 1996. Pediatrics.

[B3] Tommiska V, Heinonen K, Lehtonen L, Renlund M, Saarela T, Tammela O, Virtanen M, Fellman V (2007). No improvement in outcome of nationwide extremely low birth weight infant populations between 1996–1997 and 1999–2000. Pediatrics.

[B4] Costeloe K, Hennessy E, Gibson AT, Marlow N, Wilkinson AR, for the EPICure Study Group (2000). The EPICure study: outcomes to discharge from hospital for infants born at the threshold of viability. Pediatrics.

[B5] Vanhaesebrouck P, Allegaert K, Bottu J, Debauche C, Devlieger H, Dockx M, François A, Haumont D, Lombet J, Rigo J, Smets K, Vanherreweghe I, Van Overmeire B, Van Reempts P, for the EPIBEL Study Group (2004). The EPIBEL study: outcomes to discharge from hospital for extremely preterm infants in Belgium. Pediatrics.

[B6] National Center for Health Statistics. http://www.cdc.gov/nchs/data/statab/natfinal2002.annvol1_26.pdf.

[B7] Smith VC, Zupancic JA, McCormick MC, Croen LA, Greens J, Escobar GL, Richardson DK (2004). Rehospitalization in the first year of life among infants with bronchopulmonary dysplasia. J Pediatr.

[B8] Schmidt B, Asztalos EV, Roberts RS, Robertson CM, Sauve RS, Withfield MF (2003). Trial of Indomethacin Prophylaxis in Preterms (TIPP) Investigators. Impact of bronchopulmonary dysplasia, brain injury, and severe retinopathy on the outcome of extremely low-birrth-weight infants at 18 months: results from the trial of indomethacin prophylaxis in preterms. JAMA.

[B9] Gewolb IH, Bosma JF, Taciak VL, Vice FL (2001). Abnormal developmental patterns of suck and swallow rhythms during feeding in preterm infants with bronchopulmonary dysplasia. Dev Med Child Neurol.

[B10] Carney D, DiRocco J, Nieman G (2005). Dynamic alveolar mechanics and ventilator-induced lung injury. Crit Care Med.

[B11] Butler WJ, Bohn DJ, Bryan AC, Froese AB (1980). Ventilation by high-frequency oscillation in humans. Anesth Analg.

[B12] Marchak BE, Thompson WK, Duffy P, Miyaki T, Bryan MH, Bryan AC, Froese AB (1981). Treatment of RDS by high-frequency oscillatory ventilation: a preliminary report. J Pediatr.

[B13] McCulloch PR, Forkert PG, Froese AB (1988). Lung volume maintenance prevents lung injury during high-frequency oscillatory ventilation in surfactant-deficient rabbits. Am Rev Respir Dis.

[B14] HIFI Study Group (1989). High-frequency oscillatory ventilation compared with conventional mechanical ventilation in the treatment of respiratory failure in preterm infants. N Engl J Med.

[B15] Clark RH, Dykes FD, Bachman TE, Ashurst JT (1996). Intraventricular haemorrhage and high-frequency ventilation: a meta-analysis of prospective clinical trials. Pediatrics.

[B16] Bhuta T, Henderson-Smart DJ (1997). Elective high-frequency oscillatory ventilation versus conventional ventilation in preterm infants with pulmonary dysfunction: systematic review and meta-analyses. Pediatrics.

[B17] Cools F, Offringa M (1999). Meta-analysis of elective high frequency ventilation in preterm infants with respiratory distress syndrome. Arch Dis Child Fetal Neonatal Ed.

[B18] Thome U, Carlo WA (2000). High-frequency ventilation in neonates. Am J Perinatol.

[B19] Bollen CW, Uiterwaal CS, van Vught AJ (2003). Cumulative meta-analysis of high-frequency versus conventional ventilation in premature neonates. Am J Respir Crit Care Med.

[B20] Thome UH, Carlo WA, Pohlandt F (2005). Ventilation strategies and outcome in randomized trials of high frequency ventilation. Arch Dis Child Fetal Neonatal Ed.

[B21] Bollen CW, Uiterwaal CS, van Vught AJ (2007). Meta-regression analysis of high-frequency ventilation versus conventional ventilation in infant respiratory distress syndrome. Intensive Care Med.

[B22] Henderson-Smart DJ, Cools F, Bhuta T, Offringa M (2007). Elective high frequency oscillatory ventilation versus conventional ventilation for acute pulmonary dysfunction in preterm infants. Cochrane Database Syst Rev.

[B23] Courtney SE, Durand DJ, Asselin JM, Eichenwald EC, Stark AR (2003). Pro/con clinical debate: high-frequency oscillatory ventilation is better than conventional ventilation for premature infants. Critical Care.

[B24] Johnson AH, Peacock JL, Greenough A, Marlow N, Limb ES, Marston L, Calvert SA, for the United Kingdom Oscillation Study Group (2002). High-frequency oscillatory ventilation for the prevention of chronic lung disease of prematurity. N Engl J Med.

[B25] Van Reempts P, Borstlap C, Laroche S, Auwera JC Van der (2003). Early use of high frequency ventilation in the premature neonate. Eur J Pediatr.

[B26] Thome U, Kössel H, Lipowsky G, Porz F, Fürste HO, Genzel-Boroviczeny O, Tröger J, Oppermann HC, Högel J, Pohlandt F (1999). Randomized comparison of high frequency ventilation with high rate intermittent positive pressure ventilation in preterm infants with respiratory failure. J Pediatr.

[B27] Gerstmann DR, Minton SD, Stoddard RA, Meredith KS, Monaco F, Bertrand JM, Battisti O, Langhendries JP, François A, Clark RH (1996). The Provo multicenter early high frequency oscillatory ventilation trial: improved pulmonary and clinical outcome in respiratory distress syndrome. Pediatrics.

[B28] Papile LA, Burstein J, Burstein R, Koffler H (1978). Incidence and evolution of subependymal and intraventricular haemorrhage: a study of infants with birth weight less than 1500 gm. J Pediatr.

[B29] (1984). An international classification of retinopathy of prematurity. Pediatrics.

[B30] Srisuparp P, Marks JD, Khoshnood B, Schreiber MD (2003). Predictive power of initial severity of pulmonary disease for subsequent development of bronchopulmonary dysplasia. Biol Neonate.

[B31] Henderson-Smart DJ, Hutchinson JL, Donoghue DA, Evans NJ, Simpson JM, Wright I, for the Australian and New Zealand Neonatal Network (2006). Prenatal predictors of chronic lung disease in very preterm infants. Arch Dis Child Fetal Neonatal Ed.

[B32] Kramer MS, Platt RW, Wen SW, Joseph KS, Allen A, Abrahamowicz M, Blondel B, Bréart G, for the Fetal/Infant Health Study Group of the Canadian Perinatal Surveillance System (2001). A new and improved population-based Canadian reference for birth weight for gestational age. Pediatrics.

[B33] Roberts D, Dalziel SR (2006). Antenatal corticosteroids for accelerating fetal lung maturation for women at risk of preterm birth. Cochrane Database Syst Rev.

